# Supplementary material for: Inflammatory factors and risk of lung adenocarcinoma: a Mendelian randomization study mediated by blood metabolites
Source: Front Endocrinol (Lausanne). 2024 Aug 27;15:1446863. doi: 10.3389/fendo.2024.1446863 (PMC11384989; doi:10.3389/fendo.2024.1446863)
Supplement: Supplementary file 1 [file DataSheet1.zip › supplementary files/S2/GCST90201012/forest.pdf]

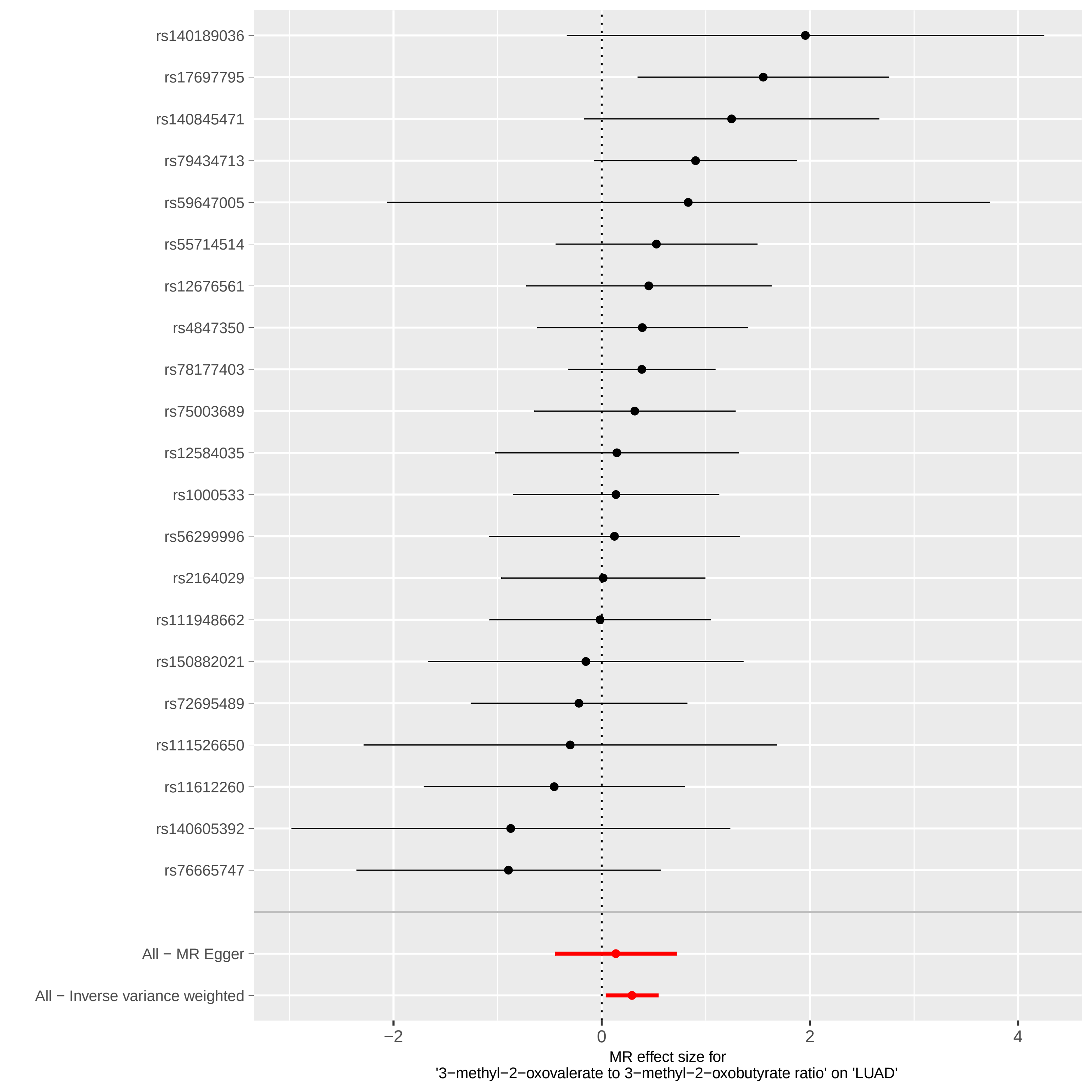
rs140189036
rs17697795
rs140845471
rs79434713
rs59647005
rs55714514
rs12676561
rs4847350
rs78177403
rs75003689
rs12584035
rs1000533
rs56299996
rs2164029
rs111948662
rs150882021
rs72695489
rs111526650
rs11612260
rs140605392
rs76665747
All − MR Egger
All − Inverse variance weighted
−2
0
2
4
MR effect size for
'3−methyl−2−oxovalerate to 3−methyl−2−oxobutyrate ratio' on 'LUAD'
